# The Oral Health Status of Chinese Elderly People with and without Dementia: A Cross-Sectional Study

**DOI:** 10.3390/ijerph17061913

**Published:** 2020-03-15

**Authors:** Sherry Shiqian Gao, Kitty Jieyi Chen, Duangporn Duangthip, Edward Chin Man Lo, Chun Hung Chu

**Affiliations:** Faculty of Dentistry, The University of Hong Kong, Hong Kong 999077, China; kjchen@hku.hk (K.J.C.); dduang@hku.hk (D.D.); hrdplcm@hku.hk (E.C.M.L.); chchu@hku.hk (C.H.C.)

**Keywords:** elderly, dementia, dental caries, periodontal diseases, toothbrushing, survey

## Abstract

Objective: The objective of this study was to compare the caries, periodontal status, and toothbrushing practices of Chinese elderly people with and without dementia. Methods: This cross-sectional study recruited Chinese people aged 65 years or over attending daycare centers in Hong Kong. The participants’ dementia status was identified from their medical record. Their demographic information and toothbrushing practices were obtained through a questionnaire survey. Caries experience, periodontal status, and oral hygiene were measured using the Decayed, Missing, and Filled Teeth (DMFT) Index, Community Periodontal Index, and Visible Plaque Index (VPI), respectively. The case matching process, using the propensity score, was conducted to match the participants in dementia and nondementia groups. The chi-square test and t-test were conducted for analysis. Results: A total of 341 elderly people participated in this study. After case matching by gender and age, 129 participants with dementia were matched with 99 participants without dementia. The mean age and mean DMFT of the dementia group versus the nondementia group were 80.9 ± 7.5 vs. 79.4 ± 6.7 (*p* = 0.428) and 22.5 ± 7.9 vs. 19.2 ± 9.3 (*p* = 0.041), respectively. There was no significant difference of periodontal status observed. The VPI of dementia and nondementia groups were 77% and 63%, respectively (*p* = 0.027). Though they had no difference in frequency of toothbrushing, more dementia participants encountered difficulties in toothbrushing than those without dementia (57% vs. 8%, *p* < 0.001). Conclusion: Compared with elderly people without dementia, Chinese elderly people with dementia had more caries experience and poorer oral hygiene in Hong Kong. They were more likely to have difficulty in performing toothbrushing.

## 1. Introduction

The World Health Organization (WHO) defines dementia as a “loss of intellectual capability of such severity as to interfere with the social or occupational functioning” [1]. Dementia is related to a broad category of brain diseases that cause an irreversible, gradual, and long-term decrease in the ability to remember and think. People with dementia are conscious and often keep normal sensory functions. However, they can suffer multiple cognitive deficits, such as the failure to recognize objects or persons, the impairment of memory, the disturbance in their executive functioning, and the deterioration of their language functions [2]. Moreover, people with dementia usually have psychiatric and behavioral changes, such as depression, agitation, and personality changes [3].

Dementia is common among elderly. It is estimated that 5% to 7% of elderly people aged 60 years or over are suffering from dementia in most regions of the world [4]. The Global Burden of Disease and Injury Incidence and Prevalence Collaborators reported that dementia affected approximately 46 million people worldwide in 2015 [5]. Because the incidence of dementia increases exponentially in the aging population, dementia will probably affect 66 million people in 2030 [4]. There is no specific test to determine if someone has dementia. Dementia is usually diagnosed based on medical history and cognitive functions. Medical imaging and blood tests can be used to rule out other possibilities [6]. Dementia can be categorized into various subtypes according to brain pathologies. The most common subtypes include Alzheimer’s disease, dementia with Lewy bodies, vascular dementia, and frontotemporal dementia [2]. Unfortunately, there is no known cure for dementia currently.

Elderly people with dementia often suffer from upper respiratory infections and pneumonia, which can be related to poor oral health. Studies reported that elderly people with dementia generally have poor oral health, including a high prevalence and risk of dental caries and periodontal disease [7,8]. A study suggested that salivary dysfunction and poor oral hygiene practice are two main reasons for their unsatisfactory oral health status [2]. Many of them have salivary dysfunction due to the side effects of their medication, such as cholinesterase inhibitors. Their resting salivary secretion is reduced because their submandibular salivary output is impaired [2]. The salivary dysfunction increases their risk of dental caries, burning mouth syndrome, and opportunistic infection, such as oral candidiasis. In addition, their oral hygiene practice is unsatisfactory, and it deteriorates with the progression of dementia [9]. Toothbrushing is the fundamental oral hygiene practice. It is important for removing dental plaque, which is the primary cause of dental caries and periodontal disease. Poor oral health also affects chewing ability and causes poor dietary intake and weight loss [10]. Oral health is an integral part of general health, and poor oral health increases the risk of systemic disease. Apart from physical health, poor oral health also adversely affects a person’s quality of life and well-being [11].

Hong Kong is a densely populated city located in Southern China with a population of more than seven million. According to “Hong Kong Population Projections 2015–2064,” the number of people aged 65 or older in Hong Kong will reach 2.6 million by 2064, which is around 36% of the whole population. As Hong Kong’s population ages, experts have warned of a looming crisis over the long-neglected problem of dementia. Hence, regular surveying is crucial and necessary for the development of evidence-based healthcare programs and evaluation of the effectiveness of those programs for reducing the disease burden. Limited updated information can be found regarding the difference of oral health of elderly with and without dementia in Hong Kong. The objective of this study was to compare the oral health status and oral health-related behaviors of Chinese elderly people with and without dementia.

## 2. Materials and Methods

This cross-sectional study was conducted in Hong Kong from March to June in 2017. Ethical approval was obtained from HKU/HA HKW Institutional Review Board (UW16-179). The reporting of the present study was in accordance with the Strengthening the Reporting of Observational Studies in Epidemiology (STROBE) statement [12].

### 2.1. Sample Size Calculation and Selection of Participants

Sample size calculation was performed by R statistical software Version 3.6.2 (The R Foundation for Statistical Computing, Austria). We estimated the difference of the mean numbers of Decayed (D), Missing (M), and Filled (F) Teeth (DMFT) between people with and without dementia was 3, and the standard deviation in both groups was 8 [2]. With the power as 0.8 and significance level as 0.05, at least 112 participants needed to be included in each of the two groups (i.e., the elderly people with dementia and the elderly without dementia).

Eight elderly daycare centers in Hong Kong were invited to join this study. The centers helped to promote this project in their daily activities or functions. A consecutive sampling technique was adopted in this study. Each center reserved one day for our team to provide dental examination, a questionnaire survey, and oral health education in the center. All the people were welcome to join the oral health education session if they were interested. Participants who met the inclusion criteria could join dental examination and questionnaire survey. The inclusion criteria were elderly people who (1) were aged 65 years or older and (2) were cooperative to dental examination. Elderly people with severe systemic diseases and poor general conditions that could not allow them to receive dental examinations were excluded. All participants or their guardians understood the aim of this study and filled in a written informed consent before they received a dental examination in the elderly center.

### 2.2. Questionnaire Survey

The demographic information (age and sex) and dementia status (yes/no) of the participants were collected from their medical record saved by the daycare centers. The participants were asked to complete a questionnaire onsite. If they were unable to fill the questionnaire by themselves, their guardians helped them answer the questions. The questionnaire explored the oral hygiene practices of the participants, including daily toothbrushing frequency, difficulty in performing self-toothbrushing, and assistance in toothbrushing. An investigator (K.J.C.) checked the completed questionnaire when it was returned and followed up those questionnaires that were missing responses or incorrectly answered.

### 2.3. Clinical Examination

Two experienced dentists (S.S.G. and D.D.) with training in community dentistry performed the clinical examinations. They were calibrated in the oral health status assessment, including oral hygiene, caries, and periodontal status. The dentists carried out the assessment using a disposable dental mirror attached to a light-emitting diode light and a ball-ended Community Periodontal Index (CPI) probe. They evaluated the dental caries experience of the elderly by the DMFT index according to the recommendation of the WHO [13]. The DT component includes all teeth with carious crown, carious root, and/or filled crown with caries. The MT component includes all missing teeth due to caries. The FT component includes teeth with filled crown but without caries. The dentists determined the periodontal status of the elderly with the CPI examination. The full dentition was divided into six sextants. Gingival bleeding, periodontal pocket, and loss of attachment were recorded by assessing the index teeth in each sextant (sextant 1—16, 17; sextant 2—11; sextant 3—26, 27; sextant 4—36, 37; sextant 5—31; and sextant 6—46, 47). The sextant with the highest score represented the periodontal status of the whole mouth [14]. The oral hygiene status was measured using the Visible Plaque Index (VPI). The dentist assessed the presence of visible plaque (yes/no) on the buccal and lingual surfaces of the index teeth (teeth 16/17, 11, 26/27, 36/37, 31, and 46/47) [15]. The VPI score was calculated as the percentage of tooth surfaces that had visible plaque. Duplicate examinations were performed on 5% of the participants at each center to determine the intra- and inter-examiner agreement.

### 2.4. Data Analysis

Two research assistants entered the data into an Excel file independently. The data was proofread before analysis. All the analysis was done by SPSS for Windows (IBM Corporation, Armonk, NY, USA). The case matching process was conducted to match the participants in dementia and nondementia groups using the propensity score. Age and sex were the two factors included in the propensity score case matching. Participants who were not successfully matched were excluded from the following analysis. Independent t-tests were conducted for analyzing continuous data between dementia and nondementia groups, including the DMFT score, VPI score, and age of the participant. Chi-square tests were conducted for analyzing categorical data between two groups, including edentulous status, periodontal status, sex, and oral hygiene practices. Cohen’s kappa statistics was used to assess the intra- and inter-examiner agreement in clinical diagnosis. The level of statistical significance was set at 0.05 for all tests.

## 3. Results

A total of 341 elderly people from eight daycare centers joined this study. After case matching by gender and age, 129 participants with dementia were matched with 99 participants without dementia. Therefore, 228 participants were included in the analysis. Most of them were female (180, 79%). The mean age of the participants was 80.2 years old. The distribution of sex and mean of age between elderly people with dementia and those without dementia are shown in Table 1. The value of kappa for caries experience (DMFT) was at least 0.90, and that for periodontal status (CPI) and oral hygiene (VPI) were at least 0.70.

Table 2 shows the dental caries and periodontal status of the elderly participants in this study. The mean DMFT score of the elderly with dementia was significantly higher than that of the elderly without dementia (22.5 vs. 19.2, *p* = 0.041). No difference was identified for the three components, i.e., DT, MT, and FT, between the two elderly groups. Almost all the elderly people with dementia (98%) had gingival bleeding. Around two-thirds of them (64%) had periodontal pockets. More than half of them (54%) had teeth with a loss of attachment of at least 6 mm. There was no difference between dementia and nondementia groups regarding the three periodontal parameters, i.e., gingival bleeding, periodontal pocket, and loss of attachment. People with dementia had a significantly higher level of visible plaque than people without dementia (77% vs. 63%, *p* = 0.027).

More than half of the participants with dementia reported difficulties when performing toothbrushing, while only a few reported so in nondementia group (57% vs. 8%, *p* < 0.001) (Table 1). More elderly people in the dementia group required caregiver-assisted toothbrushing than those without dementia (17% vs. 3%, *p* = 0.001). Four elderly people (3%) with dementia stated they did not brush their teeth daily, whereas all elderly without dementia performed toothbrushing every day. More than one-third (36%) of the elderly with dementia and 44% of the elderly without dementia had dental visits within one year, and the difference was not statistically significant (*p* = 0.241).

## 4. Discussion

Regular surveying is important for the development of evidence-based healthcare programs. This study provides updated information of the oral health status and oral health-related behaviors of Chinese elderly people with and without dementia. Dental professionals can develop and carry out a more specific oral health education program and dental care by better understanding the oral health-related situation of people with dementia. Because this cross-sectional observational study was not an epidemiological survey, the random sampling method was not adopted in this study. Instead we used the consecutive sampling technique to recruit the participants, which was simple and efficient. Consecutive sampling can lead to selection bias because the variety of participants may be limited by the same geographical region. To address this selection bias, we chose eight daycare centers located in different representative districts in Hong Kong. Elderly people with different backgrounds could join this study because the recruitment of the participants was separately conducted in eight centers.

We followed the latest WHO oral health survey recommendation [13] for selecting the age group and the examination for almost all the clinical parameters, such as caries experience, in this study. Regarding the assessment of gingival bleeding and periodontal pocket, the latest recommendation by the WHO was to examine all teeth present in oral cavity. However, the protocol of probing six sites of every tooth can cause considerable discomfort and time for assessment. Some elderly participants would withdraw from the joining the survey because they were annoyed and dissatisfied with the lengthy and uncomfortable assessment. Therefore, the investigators decided to report the periodontal status with gingival bleeding and periodontal pocket using the indexed teeth from the six sextants described by the WHO Oral health surveys: Basic methods (fourth edition) published in 1997 [14].

The results of this study showed that elderly people with dementia had a higher DMFT score than people without dementia, which is consistent with reports from other countries [7,16]. A previous study in Hong Kong also showed that the caries experience in the dementia group was higher than that in the nondementia group [2]. We also found there were more edentulous cases in elderly people with dementia than those without dementia. Several reasons can be related to their unsatisfactory dentition status. First, elderly people with dementia often suffer from upper respiratory tract infections and pneumonia, which is often related to their poor oral health [17]. Second, medication for dementia, such as cholinesterase inhibitors, can lead to salivary dysfunction. Studies reported that submandibular salivary output of patients with dementia can be impaired. The reduced resting salivary secretion renders a high caries risk among elderly people with dementia [2,18]. Last but not least, elderly people with dementia often have poor oral hygiene, and therefore more bacteria in the oral cavity, which is associated with the increased risk of dental caries development [19].

In this study, we found that the visible plaque level was significantly higher in the elderly people with dementia, which may be the reason for their higher caries experience than those without dementia. In addition, the accumulation of dental plaque offers abundant opportunities for bacteria to initiate local infections which can spread systematically. The oral bacteria can be associated with various systemic diseases, such as infective endocarditis and aspiration pneumonia by hematogenous dissemination or through inflammatory mechanisms [20,21]. Controlling the bacteria level in the oral cavity is crucial for good general health in elderly people.

We found no significant difference of the periodontal status between dementia and nondementia groups in the studied population. This result is consistent with a previous study done in Hong Kong [2]. Nevertheless, the prevalence of gingival bleeding, periodontal pocket, and loss of attachment was high in both groups, which indicated that the periodontal status in Hong Kong elderly was generally unsatisfactory. A recent study reported that porphyromonas gingivalis, which causes gingivitis, may be related to Alzheimer’s disease, which is one of the main subtypes of dementia [22]. The bacteria can move from the oral cavity to the brain. Once in the brain, it releases enzymes called gingipains. This enzyme can destroy nerve cells, which leads to memory loss and impairment of other functions, and eventually causes Alzheimer’s disease. Therefore, treatment for gingivitis should be provided to elderly people to reduce the risk of Alzheimer’s disease and further development of dementia.

Poor oral health affects chewing ability and causes poor dietary intake and weight loss. In addition, poor oral health can adversely affect a person’s quality of life, overall health, and well-being [23]. Therefore, it is crucial to maintain good oral health for the elderly, especially for those with dementia or other chronic diseases. The oral health problems that elderly people with dementia face, including dental caries, high bacteria levels, and gingivitis, are ambulatory care-sensitive conditions. One of the most important oral hygiene practices to reduce the bacteria level and improve oral health is toothbrushing. However, because people with dementia always present symptoms like impaired memory and disturbance of behaviors, it is always challenging for them to perform appropriate and effective oral hygiene practices.

Our study found that more than half of the elderly people with dementia had difficulty in performing toothbrushing, which was significantly higher than elderly people without dementia. However, only 17% of the dementia people had received assistance during toothbrushing from their caretakers. One study reported that people with dementia always need assistance with their oral hygiene care [24]. However, their family members or caretakers found that it was difficult to help them brush their teeth because of their uncooperative behaviors. Moreover, the home-based caretakers also reported that providing oral healthcare to people with dementia would add high levels of caretaker’s burden to the family [24]. Although it is difficult for the community to assist elderly people with dementia to perform oral hygiene practices, effective community care and case management is important to prevent ambulatory care-sensitive conditions arising from dental problems and to prevent the need for hospital admission, which are important indicators of the quality and efficiency of the whole health system. Therefore, dental professionals should develop specific support or training programs for the community, including family members, caretakers, and staff working in elderly homes or institutions, to provide effective assistance for the oral hygiene practices in elderly with dementia.

With the limitations of the present study, the authors suggest there can be improvements in the future studies. First, most of the participants in this study were female. The participant recruitment was performed in elderly daycare centers. It has been reported that elderly females are more likely to join social activities than males [25]. Therefore, females may attend the elderly centers more regularly and are more likely to join different programs than males. To balance the sex distribution, representative sampling may be considered in the future studies. Second, we only recorded caries experience, periodontal status, and oral hygiene status in the clinical examination because it was difficult for the elderly people with dementia to cooperate with a long-time dental examination. There may be other clinical parameters and behaviors related to elderly people with dementia, such as saliva flow rate, mucosa abnormality, and the use of mouth rinses. Further research can be considered to investigate those factors.

## 5. Conclusions

Compared with elderly people without dementia, Chinese elderly people with dementia had more caries experience and poorer oral hygiene. More elderly people with dementia experienced difficulties in toothbrushing, but only a few of them had received assisted toothbrushing by the caregiver. Community oral healthcare for elderly people with dementia can be effective in preventing ambulatory care-sensitive conditions arising from dental problems. Dental professionals should develop specific service and training programs to support the community oral healthcare.

## Figures and Tables

**Table 1:** Age, sex, toothbrushing frequency, caregiver-assisted toothbrushing, difficulty in toothbrushing, and dental visit behavior of the elderly with or without dementia.

Independent Variables	Elderly with Dementia	Elderly without Dementia	*p*-Value
Age	(n = 129)	(n = 99)	0.428
	80.9 ± 7.5	79.4 ± 6.7	
Sex	(n = 129)	(n = 99)	0.113
Male	32 (25%)	16 (16%)	
Female	97 (75%)	83 (84%)	
Toothbrushing frequency	(n = 125)	(n = 99)	0.189
None or less than once daily	4, 3%	0, 0%	
Once daily	19, 15%	14, 14%	
Twice or more daily	102, 82%	85, 86%	
Caregiver-assisted toothbrushing	(n = 126)	(n = 98)	0.001
Yes	22, 17%	3, 3%	
No	104, 83%	95, 97%	
Difficulty in toothbrushing	(n = 115)	(n = 98)	<0.001
Yes	65, 57%	8, 8%	
No	50, 43%	90, 92%	
Last dental visit	(n = 116)	(n = 88)	0.241
Within 1 year	42, 36%	39, 44%	
More than 1 year	74, 64%	49, 56%	

**Table 2:** Caries experience, edentulous status, periodontal status, and oral hygiene condition of the elderly with or without dementia.

Independent Variables	Elderly with Dementia	Elderly without Dementia	*p*-Value
Caries experience	(n = 129)	(n = 99)	
Decayed, missing and filled teeth (DMFT)	22.5 ± 7.9	19.2 ± 9.3	0.041
Decayed teeth (DT)	2.1 ± 3.1	2.3 ± 2.6	0.997
Missing teeth (MT)	16.5 ± 9.5	13.0 ± 9.2	0.528
Filled teeth (FT)	4.0 ± 4.6	3.9 ± 5.2	0.677
Edentulous status	(n = 129)	(n = 99)	0.059
Non-edentulous	115, 89%	95, 96%	
Edentulous	14, 11%	4, 4%	
Gingival bleeding (GB)	(n = 100)	(n = 92)	0.512
No	1, 1%	2, 2%	
Yes	99, 99%	90, 98%	
Periodontal pocket (PP)	(n = 100)	(n = 92)	0.478
0–3 mm	28, 28%	33, 36%	
4–5 mm	59, 59%	47, 51%	
6 mm or above	13, 13%	12, 13%	
Loss of attachment (LoA)	(n = 99)	(n = 92)	0.967
0–3 mm	13, 13%	13, 14%	
4–5 mm	32, 32%	29, 32%	
6–8 mm	40, 40%	34, 37%	
9–11 mm	10, 10%	12, 13%	
12 mm or above	4, 4%	4, 4%	
Oral hygiene condition	(n = 108)	(n = 94)	0.027
Visible plaque index (VPI)	77 ± 28 %	63 ± 33 %	
